# 

**Published:** 1849-07

**Authors:** 


### MISSISSIPPI VALLEY ASSOCIATION OF DENTAL SURGEONS.

This Society stands adjourned, to meet on the second Tuesday of September, 1849, at 10 o'clock, A. M., in Louisville, Ky.

We hope the members of the Society will bear this in mind, and also others who may wish to unite with the Society,—[CIN. ED.

---

### SUBSCRIPTIONS TO THE REGISTER.

We send bills to those who are in arrears for the Register, and hope that they will be promptly met, as the work cannot be sustained with out the means.—[CIN. ED.

---

### OUR EXCHANGES.

We would tender our thanks to the editors of the following periodicals, for the regularity with which they have been mailed to our address:

The Charleston Medical Journal and Review;
The Missouri Medical and Surgical Journal;
The Boston Medical and Surgical Journal;
The American Journal and Library of Dental Science;
The Dental Recorder;
The Dental News Letter;
The Dental Intelligencer.—[CIN. ED.

### BACK NUMBERS OF THE REGISTER.

New subscribers can be furnished with the work from its commencement. We have, however, but a few copies of the first number, Vol. I, on hand. Those who subscribe, and have received this, will please let us know.—[Cin. Ed.

---

### DELAY IN THE PUBLICATION OF THE REGISTER.

The present number of the Register has been delayed for near a month, in consequence of the sickness in our city. We feel that this is of itself ample apology, and will be appreciated by our subscribers. —[Cin. Ed.

---

### MANUFACTURERS OF TEETH

Will remember that the Mississippi Valley Association of Dental Surgeons, award premiums for the best specimens of teeth presented at their next annual meeting; which will be held on the second Tuesday of September next, in Louisville, Ky.—[Cin. Ed.

### INDEX TO VOLUME II.

SELECTIONS AND COMMUNICATIONS.

Anchylosis of the Jaw, .......... 89
Acute Inflammation of the Sublingual Glands—Dr. Boykin, .......... 97
Arrangement of Teeth—Dr. Goddard, .......... 132
Address to Grad. Class Baltimore Col. Den. Sur.—Dr. Parmly, .......... 138
Articulating Sets of Teeth—Dr. Peebles, .......... 141
Alveolar Abscess—Dr. J. Taylor, .......... 166
Andral on Human Fluids, .......... 173
Antiquity of Anæsthetic Agents, .......... 180
Clergymen and Physicians as Dentists—Dr. Power, .......... 53
Clark on Pivot Teeth, .......... 142
Du Bouchet's Nostrum—Dr. Berry, .......... 96
Extirpation of Os Maxillare Superius, .......... 184
Fourth Annual Meeting Miss. Val. Ass. Dent. Surg. .......... 18
From my Note Book—Dr. Whitney, .......... 68
Facial Neuralgia, .......... 185
Gangrene of the Mouth in Children, .......... 186
Gum Boils, &c., .......... 191
Hæmorrhage, observations on—Dr. Brown, .......... 1
" after removal of Canine Root, .......... 66
" from extracting teeth, and Dental Fistulæ, .......... 187
Importance of Medico-dental Education, .......... 77
Injurious Effects of Chloroform, .......... 196
Lamp, the—Dr. Taft. .......... 94
Latest Humbug, .......... 100
Letter from Dr. Stratton, .......... 40
" " Dr. Haven, .......... 43
" " Prof. Powell, .......... 58
" " Dr. Whitney, .......... 75
" " Dr. Peebles, .......... 105
Lawrence's Blow pipe, .......... 103
Maxillary Abscess—Dr. Griffith, .......... 137
Mesmerism and Dental Surgery, .......... 181
Meteorological Tables, .......... 211, 212, 213, 214
New Amalgam, Evans' .......... 176
" " " Remarks on—J. L. Levison, .......... 178
Pivot Teeth—Dr Bonsall, .......... 25
Preservation of Teeth—R. T. Willard, .......... 157
Preperation of Wood Pivots, .......... 164
Proposed Test for Detecting Impure Chloroform, .......... 197
Progress and Decline of the Epidemic, .......... 209
Quack Dentists, .......... 161
Resolutions of Dental Class Ohio Col. Den. Surg., .......... 152
Synopsis of Discussions of Miss. Val. Ass. Den. Surg., .......... 28

Strictures—W. A. Pease, D. D. S.....83
Salivary Calculus taken from a Horse,.....104
Third Denuition,.....70
Tumors of the Face,.....190
Tic Doloreux—Dr. Hullihen,.....122
Use of the Key—Dr. Taft,.....35
Ulcers of the Tongue,.....195
Voluntary Associations—Dr. Brown,.....61
Valedictory Address—Dr. J. Tavlor,.....109

EDITORIAL.

Amer. Jour. and Library of Dental Science,.....51, 107
Agents for Dental Register,.....107
Ambler's Journal of Dental operations,.....156
Back numbers of the Register,.....208
California Fever,.....204
Contributions to Ohio College Dent. Surgery,.....204
Cholera,.....106, 206
Dental Advertisements,.....46
Dental and Medical Surgery,.....155
Delay in the pnblication of the Register,.....208
Great Effects and Little Causes,.....198
General Index to Vol. I,.....50
Hill's Stopping,.....45
Harris' Dictionary of Dental Science,.....202
Hill's Compound for filling teeth,.....205
Impressions in Plaster, Wax, and Gutta Percha,.....145, 150
Manufacturers of Teeth,.....208
Method to prevent plate springing in soldering,.....207
Miss. Val. Ass. Dental Surgeons,.....206
Modified Tooth Key,.....47
Medical notice,.....201
Notice to Manufacturers,.....105
N. Y. Dental Recorder,.....48
Our Exchanges,.....206
Obituary of Dr. Hunt,.....51
Professional Gossip,.....151
Register, the.....205
Rising in the World,.....201
Subscriptions to the Register,.....206
Students and Graduates of Ohio Col. Den. Surg.,.....153
Steam in Dentistry,.....156
Third Dentition, Vicar Mens.,.....50
To Correspondents,.....50
To Subscribers,.....51
Van Emen's Bellows and Blow pipe,.....199
White's tooth ache drops,.....49

